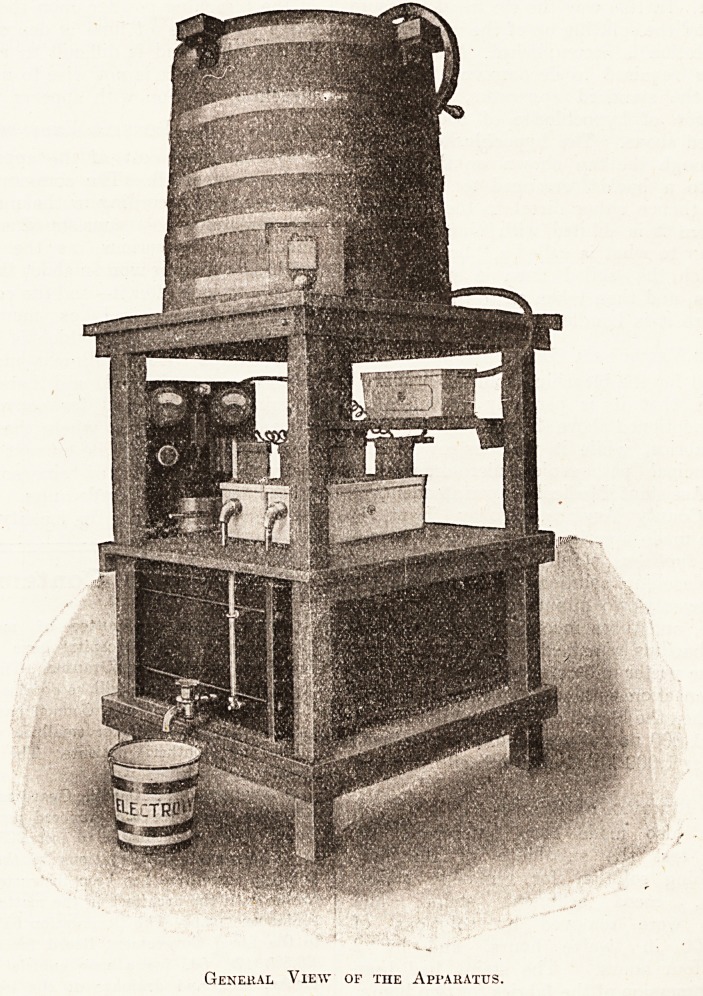# Bleaching and Disinfecting by the Aid of Electrolysis

**Published:** 1915-01-16

**Authors:** 


### BLEACHING AND DISINFECTING BY THE AID OF ELECTROLYSIS.

### Experiments at Bexley Mental Hospital.

The bleaching and disinfecting properties of chlorine have lately been adapted by a new apparatus which merits the attention of institutional managers. By the aid of an electric current electrolytically decomposing a solution of ordinary common salt, a solution of hypochlorite of soda is produced of such a strength that it can be employed, when diluted with nine times its volume of water, in the ordinary washing-machines. A certain quantity of the very cheapest salt, according to the manufacturers, is dissolved in the vat shown at the top of the illustration. It is usual to dissolve the salt the night before, so as to allow of settlement of foreign matter, and the solution is filtered before being used. The solution in the vat has a strength of 4 per cent. of sodium chloride. When the apparatus is to be used, the solution is allowed to pass down from the vat through the curved pipe, shown to the right of the drawing, into a small cistern, where it is filtered, and from which it is fed into the electrolytic tanks.

General View of the Apparatus.

One important feature in connection with the electrolytic tanks should be noted here. They are arranged to be worked from the ordinary 220-volt continuous current electrical supply service. Very small pressures are usually employed in chemical reactions, but, by a special arrangement of what are virtually cells in the electrolytic tank, 110 volts is used up, and, by putting two electrolytic tanks in series, causing the current to pass through them one after the other, the whole 220 volts is absorbed. The electrolytic tanks are divided up into sections by glass plates and by carbon plates, and the brine solution is allowed to flow continuously through the tanks. It is arranged that the solution passes first over one glass plate and then under the next; first over one carbon plate and then under the next. In this way the electrical pressure is absorbed, and the splitting up of the whole of the salt in the solution is accomplished. A current of 10 amperes is required to decompose 12 gallons per hour in the standard apparatus, the result being a solution of hypochlorite of soda of the strength named above. The hypochlorite solution flows out through the tap shown on the left of the figure into a bucket arranged to receive it, and is taken thence either direct to the washing-machine, where it is diluted with nine times its own volume, or to what is called a " breakdown " tank, in which the soiled linen is steeped for fifteen minutes, and then taken on to the washing-machine, where it is handled in the usual way.

### Its Value as a Germicide.

It is claimed for the bleaching solution produced by electrolysis that it is perfectly harmless in the strength mentioned above (3 grammes per litre) to ordinary fabrics, while it will destroy at any rate the principal pathogenic micro-organisms. Dr. Faulks, Assistant Medical Superintendent at the London County Asylum, Bexley, Kent, is stated to have made a series of tests upon *B. coli communis*, *B. typhosus*, staphylococci, and streptococci. The bleaching solution killed the *bacillus typhosus* in twenty-two minutes with an 0.5 per 1,000 mixture, and tubes inoculated from hanging drops of the bacillus remained sterile after thirty-six hours. The writer understands that Dr. Faulks reports, in connection with cultures of *B. coli communis*, that when the bleach culture contained more than 0.3 per 1,000 no cultures could be obtained; that the 0.3 per 1,000 killed all the bacilli, but not the cocci.

### Removal of Stains.

Dr. Faulks also made some interesting experiments to test the suitability of the electrolytic bleaching solution for removing stains. Blood stains, cocoa sediment stains, and fæcal stains were removed by solutions of 0.5 per 1,000 in twelve hours, and by 2 per 1,000 in four hours. The stains had been produced by immersion of the fabrics for two hours, followed by a drying for four days. Dr. Faulks found that immersion of any white fabric in a solution of 2 per 1,000 of the electrolytic bleaching for half an hour removed stains of every kind, while in four hours the fabric was quite clean. It should be noted that the electrolytic bleaching solution cannot be used for coloured fabrics, as it would remove the dye.

Dr. Faulks also made some exceedingly interesting experiments to determine the destructive effect of the bleaching solution upon different fabrics, and he found that for practical purposes it was negligible. This latter, it need hardly be mentioned, is a most important point. There are a number of bleaching solutions and bleaching powders upon the market, which are reported to be used in public laundries, and it is the common belief that a good deal of the destruction of clothes which goes on in laundries is due to the action of the bleaching powder upon the fabric. In some cases carbonate of lime is deposited in the pores of the fabric and is difficult to remove. The one substance that it is not wise to use the electrolytic bleaching solution with appears to be silk.

### Running Expenses.

The running cost of the apparatus is claimed to be very small. The commonest form of salt can be used, according to the manufacturers, and the remaining cost consists of attendance—which should not be serious, as the engineer, or any intelligent man or woman about the laundry, should be able to look after it—and the cost of the current. The current required, as mentioned above, is 10 amperes, 220 volts, which is roughly $2\frac{1}{5}$ units. It is always wise to allow a margin for leakage in matters of this kind, and there will be a further margin to be allowed for converting alternating current to continuous, where the local supply service is alternating. The current per day for electrolysing 120 gallons of brine solution should not exceed 30 Board of Trade units. The cost of these will vary according to the locality.

## Figures and Tables

**Figure f1:**